# Does disaster-related relocation impact mental health via changes in group participation among older adults? Causal mediation analysis of a pre-post disaster study of the 2016 Kumamoto earthquake

**DOI:** 10.1186/s12889-023-16877-0

**Published:** 2023-10-11

**Authors:** Yoko Matsuoka, Maho Haseda, Mariko Kanamori, Koryu Sato, Airi Amemiya, Toshiyuki Ojima, Daisuke Takagi, Masamichi Hanazato, Naoki Kondo

**Affiliations:** 1 Department of Health and Social Behavior, The University of Tokyo, Bunkyo-ku, Tokyo, Japan; 2 Department of Social Epidemiology, Kyoto University, Kyoto-shi, Kyoto, Japan; 3 Center for Preventive Medical Sciences, Chiba University, Chiba-shi, Chiba Japan; 4 Department of Community Health and Preventive Medicine, Hamamatsu University School of Medicine, Hamamatsu, Shizuoka Japan; 5 Department of Social Epidemiology, Graduate School of Medicine, Faculty of Medicine, Science Frontier Laboratory, Kyoto University, Floor 2, Yoshida-konoe-cho, Sakyo-ku, Kyoto-shi, Kyoto, Japan

**Keywords:** Earthquake, Disaster, Relocation, Older adults, Group participation, Japan

## Abstract

**Background:**

Disaster-related relocation is associated with depression and post-traumatic stress disorder, especially in older adults. Disaster-related relocation often deprives survivors of opportunities for social group participation, potentially deteriorating their mental health. On the contrary, the relocation could also be an opportunity for optimizing social relationships, ending/reducing unwanted participation. This study examined the potential mediation effects of changing participation for the link of disaster-related relocation to mental health.

**Methods:**

We analyzed a pre-post disaster dataset of functionally independent older adults from the Japan Gerontological Evaluation Study. Following the 2013 survey, a follow-up survey was conducted seven months after the 2016 Kumamoto earthquake (n = 828).

**Results:**

The causal mediation analyses indicated that compared to no relocation, the relative risk for experiencing major depressive episodes among those relocating to temporary housing was 3.79 [95% confidence interval: 1.70–6.64] (natural direct effect). By contrast, the relative risk for those renewing (either ceased or started) group participation was 0.60 [95% CI: 0.34–0.94] (natural indirect effect).

**Conclusions:**

Optimization of social ties according to a renewal of group participation status might have protected older adults in temporary housing against depression.

**Supplementary Information:**

The online version contains supplementary material available at 10.1186/s12889-023-16877-0.

## Background

Natural disasters, such as earthquakes, can cause long-lasting impairments in survivors’ mental health [1–6]. Although most people affected by disaster recover from mental illnesses within one year, some persist for several years afterward [2, 5]. Experiencing a natural disaster is particularly harmful to vulnerable populations, such as older adults, due to their limited ability to respond to disaster-induced changes in the living environment [7–9]. Notably, disaster-related relocation could increase the risk of depression [10–16] and posttraumatic stress disorder (PTSD) [1, 15, 17, 18].

Disaster-related relocation leads to changes in social relationships [19], such as participation in community group activities (e.g., volunteering, sports, or hobby groups). However, the results may differ by relocation type. For example, after the Great East Japan Earthquake of 2011, those who relocated to temporary public housing through a governmental group relocation program had more frequent social group participation. Moreover, they had richer social interactions with friends, compared to those who relocated to the temporary housing via lottery, or to other independently sourced housing [20]. The relocation program supported community-based group relocation and aimed to maintain pre-existing social ties among disaster survivors from the same community. Thus, it may offer opportunities for participation in social groups, which is considered an activator of social networks or engagement [21–23]. Through group participation, individuals strengthen their social ties with acquaintances [21] or form unanticipated ties with new people [24]. In some previous studies, group participation after disasters reduced the depression severity of survivors [25, 26].

Therefore, the impacts of disaster-based relocation on mental health can be partially mediated by changes in group participation. This study aimed to clarify whether changes in group participation mediated disaster-related relocation and older adults’ mental health problems. Older adults often have difficulties adjusting to new environments. Thus, any changes before and after relocation could constitute environmental stressors for them [27, 28], including the cessation of group participation—or even starting to participate in a new group. However, this phenomenon has been previously overlooked in the research. Hence, we hypothesized that disaster-related relocation may increase the risk of mental health issues in older adults; moreover, changes in group participation, as additional stressors, may mediate the negative impact of relocation. We also hypothesized that the effects may differ according to relocation type. Specifically, compared to individual relocation, group relocation (to temporary housing) may be less stressful, and consequent changes in group participation would be less severe as stressors. This is because the group relocation program may bring acquaintances from pre-disaster communities.

## Methods

### Design, settings, and participants

**Fig. 1:**
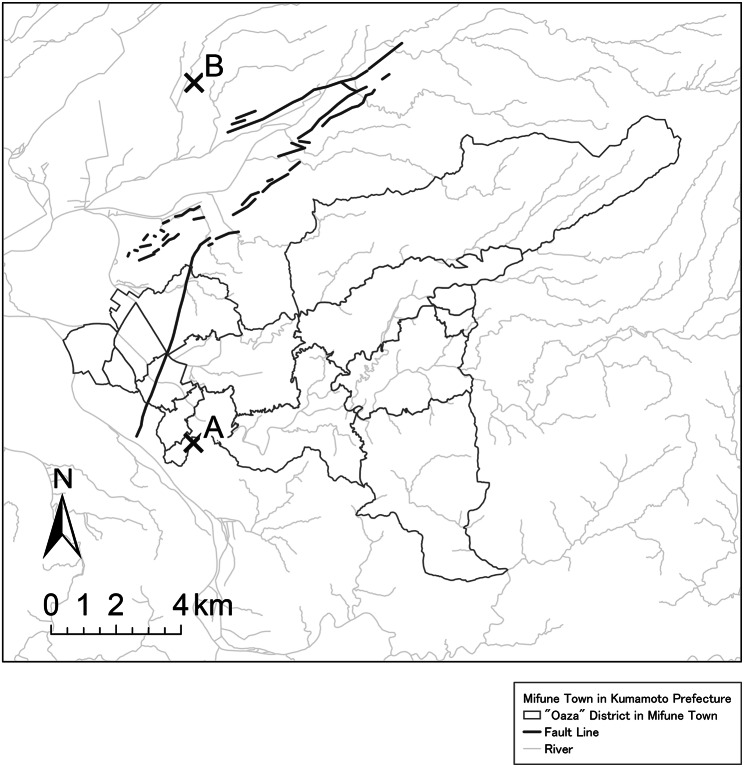
Map of Mifune Town, Kumamoto Prefecture in Japan (2013–2016). Point A is the epicenter of the Kumamoto earthquake on April 14, 2016. Point B is the epicenter of the Kumamoto earthquake on April 16, 2016 [36]

We utilized longitudinal data from the Japan Gerontological Evaluation Study (JAGES), a cohort study [29, 30]. The study site was Mifune Town in Kumamoto Prefecture (Japan) (Fig. 1). The total population of Mifune Town was 17,237 people from 6,317 households, and the aging rate was 31.6% (5,440 were aged ≥ 65 years) in the 2015 census year [31]. In April 2016, Kumamoto and nearby prefectures were hit by earthquakes and consecutive aftershocks. There were two major earthquakes of magnitude (Mw) 6.2 and 7.2 on April 14 and 16, respectively [32]. Consequently, in Mifune Town, seven people died, 4,640 houses were damaged, and 6,191 people were evacuated [33]. Moreover, a flood occurred on June 20, 2016, in which 66 houses were damaged or inundated [34]. We examined a case of older adults affected by the 2016 Kumamoto earthquake. At the time, the socio-physical environment of temporary housing was improved by prefectural-level administrative efforts based on lessons from the Great East Japan Earthquake of 2011. Specifically, the Kumamoto prefectural government established building standards for temporary housing (called “Kumamoto Type Default”) [35]. They designated public gathering areas in temporary housing complexes. A support center consisting of a council of social welfare and non-profit organizations was established, and life supporters were dispatched mainly to temporary housing complexes to talk with disaster survivors, encourage them to join events at public gathering places, and link them to professionals. Local governments in the affected areas also implemented a group relocation program for temporary housing occupants. We presumed that the improved temporary housing sites after the 2016 Kumamoto earthquake may benefit the residents’ mental health.

We employed a pre-post disaster dataset of Mifune Town using the mail-based questionnaire survey of the JAGES datasets of 2013 (in October, 30 months pre-earthquake) and 2016 (in November, seven months post-earthquake). We also connected the geographical and demographic information (area slope and population density calculated by district levels) [37] to the dataset. The 2016 Kumamoto earthquake and flood hit between the two waves of the JAGES survey, conducted every three years. The pre-post disaster dataset thus allowed us to estimate their effects with minimum recall bias.

The JAGES population comprised functionally independent adults (aged ≥ 65 years) without certification of long-term care needs. We limited the data to those who lived in Mifune Town in 2013 and 2016 and responded in both waves. Respondents who had invalid data regarding gender and/or age, responses that were lost to follow-up, or had no baseline response were excluded.

### Outcomes

We tested for two mental health issues—major depressive episodes (MDE) and PTSD symptoms—using the Screening Questionnaire for Disaster Mental Health (SQD) [38, 39]. The SQD was developed based on the Post-Traumatic Symptom Scale [40] and the Diagnostic and Statistical Manual of Mental Disorders, Fourth Edition [41]. The SQD comprised nine items on PTSD (SQD-P) and six items on MDE (SQD-D). These were validated against the Clinician-Administered PTSD Scale [42] and the Structured Clinical Interview for the revised Diagnostic and Statistical Manual of Mental Disorders, Third Edition, Major Depression Sect. [43], respectively [39]. The receiver operating characteristic curves and their standard errors (SEs) for the SQD-P and SQD-D were 0.91 (SE = 0.04) and 0.94 (SE = 0.03) [39], respectively. Based on the guideline, we set the cutoff for PTSD symptoms as an SQD-P score of ≥ 5, which included at least one symptom of intrusion, and for MDE, an SQD-D score of ≥ 4, with either depressed mood or diminished interest [38]. For a sensitivity analysis, we used the Japanese version of the 15-item Geriatric Depression Scale (GDS-15) [44–46] with a cutoff of ≥ 5 for another screening for depression [47, 48]. All the outcomes were binary variables measured during the second wave in 2016 after the earthquake.

### Exposure

Exposure was a categorical variable of three states, based on the responses to the second wave: relocation to temporary housing, relocation to other housing types, and no relocation after the 2016 Kumamoto earthquake or floods in 2016. Temporary housing built after the earthquake was opened for affected people on June 5, 2016 [49]. Other types of housing used included public rental housing, private rental housing, privately owned houses, or others. Based on personal communication with the government and public health staff members of Mifune Town, the town implemented a group relocation policy for those who were relocated to temporary housing. They also established public gathering areas for each temporary housing complex and dispatched life supporters from the support center to help disaster survivors. Therefore, in our study site, we viewed relocation to temporary housing as *group relocation*, and relocation to other housing types (such as private housing) as *individual relocation*.

### Potential mediators

We set variables of changes in group participation as potential mediators. We defined group participation when a person participated in any one of the following at least a few times a year: volunteer groups, sports groups or clubs, hobby activity groups, senior citizen clubs, community associations, study or cultural groups, nursing care prevention activities, or activities that taught skills or passed experiences to others [50]. We defined the state as “ceased” if one participated in any social group in the first wave and no longer participated in the second wave after the 2016 Kumamoto earthquake. Inversely, we defined the state as “started” if one did not participate in any group in the first wave and joined any group in the second wave. Finally, if one continued participating or not participating in any group between the two waves, we defined the state as “sustained.” In our main analyses, we regarded ceasing or starting group participation as canceling or obtaining a membership to any one of the groups. For a sensitivity analysis, we adopted a different potential mediator—specifically, the change in the frequency of group participation. We utilized a binary variable to present increased or decreased frequency of group participation compared to the cutoff of “once a month.” The alternative mediator indicated a change in frequency if the frequency of participation reduced from at least once a month to less than once a month, and vice versa.

### Covariates

We included the covariates of the baseline personal or regional characteristics from the 2013 survey, referring to existing studies on the effects of disasters on mental health. Baseline personal characteristics included gender [51], age [52, 53], equivalent household income (low income: < 2.0 million yen; below the mean of older adults’ households in 2013) [54], years of education (< 9 years of compulsory education indicated low education) [16, 53], lived alone or not, had an illness or not [53, 55], had a job or not [52], had group participation or not, and had depressive symptoms or not. Depressive symptoms were based on cutoffs of GDS scores as not depressed (0–4), moderately depressed (5–9), and depressed (10–15) [48]. Baseline regional characteristics included the standardized score of population density (persons/km^2^) and the standardized score of area slope (%) [37]. Data on population density and area slope were measured at the district (*oaza*) level of the town. We displayed these regional characteristics on maps at the district level, using ArcGIS Pro 2.8 (Esri, Redlands, CA, USA) (Additional Figs. 1 and 2 [see Additional file 1]). As shown in the maps, the western part of the town was flatter with a higher population density, and the eastern part was more mountainous with a lower population density. We also adjusted for disaster damage based on data obtained from the follow-up survey in 2016, such as housing damage based on the administrative criteria (“totally collapsed” vs. “almost collapsed” vs. “half collapsed” vs. “minor damage” vs. “no damage”) and farmland damage based on self-report (“severely damaged” vs. “partially damaged” vs. “no damage or no farmland”).

**Fig. 2:**
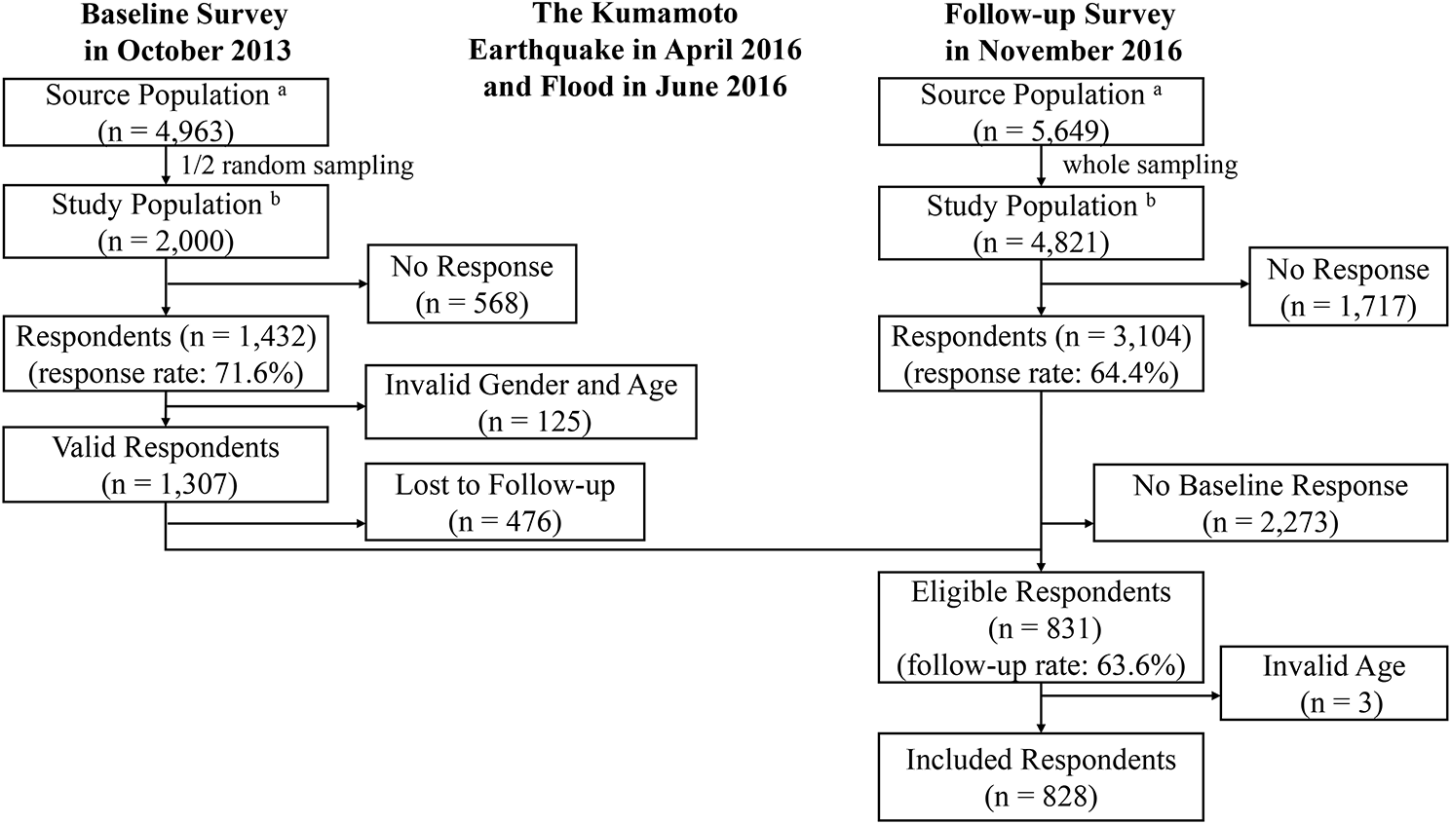
Flowchart of the selection of the analytic sample (n = 828), Mifune, Japan (2013–2016). The source population ^a^ contains older adults (≥ 65 years old) from Mifune during each survey year. The study population ^b^ contains older adults (≥ 65 years old) of the source population, without certification of long-term care needs, from Mifune during each survey year

### Statistical methods

**Fig. 3:**
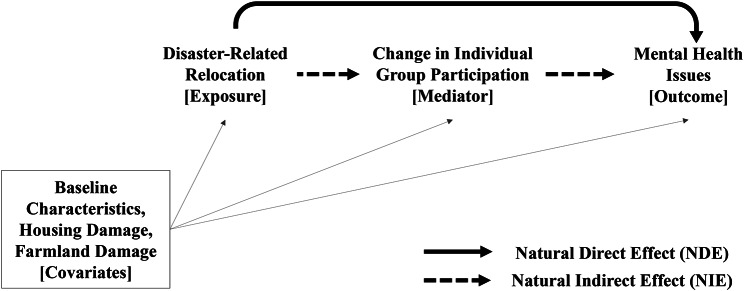
Hypothetical paths tested and the variables modeled in this study for the respondents included in the analyses (n = 828), Mifune, Japan (2013–2016)

The hypothetical causal model is summarized in Fig. 3. We assumed that in this causal model, baseline covariates, exposure, potential mediators, and outcomes would be consistent with the chronological order and that there would be no exposure-induced mediator-outcome confounders or unmeasured confounding [56, 57]. The variables other than baseline covariates were measured at the same wave (second). However, as mentioned above, the relocation started at least five months before the second wave, around the time when temporary housing opened. Additionally, we assumed that changes in group participation (i.e., “ceased” or “started” by quitting or newly joining social groups) occurred right after disaster-related relocation as the result of disruption of community structures induced by displacement of people [19, 20]. Moreover, given that the onset of mental health issues does not necessarily immediately follow disasters [2, 5], we assumed that people may develop mental health issues after experiencing multiple stressors induced by disasters. Therefore, we hypothesized that disaster-related relocation preceded changes in group participation, and mental health issues followed them.

We conducted a first-leg analysis and regression analysis of potential mediators (changes in group participation) on exposure (relocation type) to confirm whether a similar trend would be observed as in a previous study [20]. We tested a multinomial logistic regression for the categorical variable of “ceased” vs. “started” vs. “sustained” group participation and Poisson regression for the binary variable of “renewed” (either “ceased” or “started”) vs. “sustained” group participation.

Next, we conducted causal mediation analyses based on the inverse odds ratio-weighted method [58, 59]. This method has been applied in examining mediators of relocation effects of Moving to Opportunity projects on adolescents’ health in the U.S. [58, 60–62]. Several aspects of the model or variable selection were suitable for our analyses (Web Appendix 1 [see Additional file 1]). We applied this method to clarify the mediators of disaster-related relocation on older adults’ mental health. We referred to a practical guideline [58] and adopted the inverse odds weight for the analyses. We calculated weights using multinomial logistic regression for each relocation type (the categorical exposure). Next, the weights were applied in the Poisson regression to derive the effect estimates (details are described in Web Appendixes 2–3 [see Additional file 1]). Overall, we approximated the estimates of the effects of relative risk (RR) for Poisson regressions [63] and bootstrapped the effect estimates 1,000 times to derive a bias-corrected 95% confidence interval (CI) [58]. The estimates included the total effect (i.e., an overall change in a counterfactual outcome due to a change in exposure from reference to another level) [56, 64] and the natural direct effect (i.e., a change in a counterfactual outcome due to a change in exposure, if a mediator did not intercept) [64–66]. We derived these effect estimates from unweighted and weighted Poisson regressions, respectively [58]. Subsequently, by log-scale calculation, we estimated the natural indirect effect (i.e., a change in a counterfactual outcome due to exposure via a change in a mediator) [56, 64–66] by subtracting the coefficient of the natural direct effect estimate from the coefficient of the total effect estimate. This is based on the assumption that the total effect could be the sum of the natural direct effect and the natural indirect effect in the counterfactual-based approach [57–59, 64]. We included a binary variable, “renewed” (either ceased or started) group participation, as the mediator, which referred to the “sustained” state. We integrated “ceased” and “started” into one category (“renewed”) to avoid ceiling or floor effects of the variables, given that those who participated in no group at the baseline could not “cease” group participation and those who already participated in any group could not “start” it. Thus, we tested how changes in group participation mediated the relationship between relocation and mental health. We set the no relocation group as a reference and compared by relocation type (relocation to temporary housing vs. other housing types, which equals group relocation vs. individual relocation in this study).

For sensitivity analyses, we tested models that included one mediator each of “ceased” or “started” group participation separately in mediation analyses to examine directionalities. Moreover, we conducted a mediation analysis for depression measured by GDS ≥ 5 including the moderately depressed status, and the alternative mediator of “change in frequency” with the “once a month” cutoff for group participation.

For all the analyses, we used the cohort dataset where variables included in the regressions were imputed by multiple imputation by chained equation (MICE). We assumed missing at random and utilized 20 imputed datasets by MICE. All the analyses were conducted using STATA version 14.2 (StataCorp, College Station, TX, USA).

## Results

Mifune Town distributed questionnaires to half of the randomly sampled population during the baseline in 2013 (n = 2,000) and to the whole study population in 2016 (n = 4,821). The response rate was 71.6% (n = 1,432) in 2013 and 64.4% (n = 3,104) in 2016. The data of 828 respondents were analyzed (Fig. 2).

[Figure 2 here]

The summary statistics of the respondents included in the analyses by relocation type before imputation are shown in Table 1. For those who relocated to temporary or other types of housing, or those who did not relocate, the proportions of MDE were 34.0% vs. 23.0% vs. 10.2%, and those of PTSD symptoms were 34.0% vs. 36.5% vs. 20.1%, respectively. In terms of change in group participation, the proportions of those who ceased group participation were 5.7% vs. 10.8% vs. 4.5%, and the proportions of those who started group participation were 5.7% vs. 2.7% vs. 4.4%, respectively.

**Table 1:** Participants’ summary statistics (n = 828) by three types of relocation exposure (before imputation)

	Types of relocation exposure (missing: n = 173)												Total ^a^ (n = 828)				
	No relocation (n = 528)				Relocation to temporary housing (n = 53)				Relocation to other types of housing (n = 74)								
	n	%	Mean	SD	n	%	Mean	SD	n	%	Mean	SD	n	%	Mean	SD	
**Outcomes**																	
MDE	54	10.2			18	34.0			17	23.0			108	13.0			
PTSD symptoms	106	20.1			18	34.0			27	36.5			183	22.1			
Depression (GDS ≥ 5)	81	15.3			17	32.1			18	24.3			148	17.9			
**Potential mediators**																	
Change in group participation:																	
Renewed (ceased)	24	4.5			3	5.7			8	10.8			43	5.2			
Renewed (started)	23	4.4			3	5.7			2	2.7			36	4.3			
Sustained	251	47.5			20	37.7			29	39.2			348	42.0			
**Covariates**																	
*Baseline personal characteristics (before earthquake)*																	
Gender:																	
Women	281	53.2			30	56.6			50	67.6			467	56.4			
Men	247	46.8			23	43.4			24	32.4			361	43.6			
Age:																	
65–69	156	29.5			11	20.8			20	27.0			230	27.8			
70–74	177	33.5			19	35.8			25	33.8			259	31.3			
75–79	99	18.8			12	22.6			15	20.3			173	20.9			
80–84	70	13.3			8	15.1			8	10.8			112	13.5			
85+	26	4.9			3	5.7			6	8.1			54	6.5			
Low income	264	50.0			28	52.8			33	44.6			422	51.0			
Low education	206	39.0			23	43.4			26	35.1			338	40.8			
Living alone	53	10.0			3	5.7			9	12.2			88	10.6			
No illness	67	12.7			7	13.2			14	18.9			108	13.0			
No job	365	69.1			34	64.2			47	63.5			557	67.3			
No group participation at baseline	97	18.4			11	20.8			14	18.9			152	18.4			
Depressive symptoms at baseline:																	
Not depressed	371	70.3			35	66.0			54	73.0			568	68.6			
Moderately depressed	61	11.6			11	20.8			6	8.1			96	11.6			
Depressed	17	3.2			1	1.9			2	2.7			25	3.0			
*Baseline regional characteristics (before earthquake)*																	
Population density (person/km^2^) ^b^	528		3280.6	862.5	53		3396.1	745.7	74		3464.4	792.6	828		3289.0	847.5	
area slope (%) ^b^	528		14.3	9.0	53		12.3	9.3	74		10.0	7.0	828		14.3	9.1	
*Disaster damage (after earthquake)*																	
Housing damage:																	
No damage	90	17.0			3	5.7			2	2.7			135	16.3			
Minor damage	233	44.1			1	1.9			6	8.1			296	35.7			
Half collapsed	171	32.4			23	43.4			21	28.4			238	28.7			
Almost collapsed	23	4.4			9	17.0			15	20.3			52	6.3			
Totally collapsed	6	1.1			17	32.1			29	39.2			56	6.8			
Farmland damage:																	
No damage/no farmland	304	57.6			22	41.5			36	48.6			407	49.2			
Partially damaged	64	12.1			7	13.2			6	8.1			89	10.7			
Severely damaged	37	7.0			7	13.2			8	10.8			58	7.0			

Abbreviations: SD, standard deviation

^a^ Total number (n) is not equal to the summation of the left three columns because it includes participants with missing relocation exposure

^b^ Variables are standardized after multiple imputations to avoid multicollinearity and for ease of interpretation

The first-leg analysis showed that relocation to temporary housing and other housing types were positively associated with ceased group participation compared to no relocation (Additional Table 1 [see Additional file 1]). Similarly, relocation to other housing types was negatively associated with started group participation. Renewed (either ceased or started) group participation was positively associated with relocation to other housing types.

The main results of the mediation analyses showed that for relocation to temporary housing, the RR for the natural indirect effect estimate of relocation via renewed (“ceased” or “started”) group participation on MDE was 0.60 [95% CI: 0.34–0.94] (Table 2). The RR for the natural direct effect estimate of relocation on MDE was 3.79 [95% CI: 1.70–6.64]. No clear associations were observed for the outcome of PTSD symptoms. Additionally, no clear associations were observed regarding relocation to other housing types.

**Table 2:** Indirect, direct, and total effect estimates of relocation on major depressive episodes; posttraumatic stress disorder

	MDE			PTSD symptoms		
	RR	95% CI ^a^		RR	95% CI ^a^	
1: Relocation to temporary housing						
Natural indirect effect (via renewed group participation)	0.60	0.34	0.94	0.70	0.38	1.16
Natural direct effect (of relocation)	3.79	1.70	6.64	2.02	0.90	3.56
Total effect	2.28	1.36	3.81	1.41	0.86	2.13
2: Relocation to other types of housing						
Natural indirect effect (via renewed group participation)	0.97	0.62	1.95	1.05	0.70	1.57
Natural direct effect (of relocation)	1.62	0.59	3.16	1.44	0.77	2.30
Total effect	1.58	0.91	2.54	1.50	1.03	2.21

Abbreviations: MDE, major depressive episodes; PTSD, posttraumatic stress disorder; RR, relative risk; CI, confidence interval

NOTE: Adjusted for the baseline covariates of gender, age, equivalent household income (< 2.0 million yen: low income), years of education (≤ 9 years of compulsory education indicated low education), lived alone or not, had an illness or not, had a job or not, with group participation or not, had depressive symptoms or not, standardized score of population density (persons/km^2^) and standardized score of area slope (%), and disaster damage in 2016 such as housing and farmland damage

^a^ Bootstrapped 1,000 times, 95% CI displays bias-corrected confidence interval

Dataset: Imputed dataset by multiple imputations by chained equation (m = 20), including an outcome (MDE or PTSD symptoms), an exposure, a mediator, and covariates as imputed variables

For the sensitivity analyses, we included the mediators of each of ceased or started group participation separately for the outcomes of MDE (Additional Table 2 [see Additional file 1]), and PTSD symptoms (Additional Table 3 [see Additional file 1]). The results of this separate mediator analysis were not comparable with the main result (Table 2) owing to the differences in references. However, we found that both ceased and started group participation showed a clear natural indirect effect estimate that could attenuate the risk of MDE and a natural direct effect estimate that could increase the risk of MDE regarding relocation to temporary housing. Similarly, no clear associations were observed regarding the outcome of PTSD symptoms and relocation to other housing types.

Moreover, the result of another mediation analysis that used the GDS as an alternative measure of depression (GDS ≥ 5) (Additional Table 4 [see Additional file 1]), showed that for relocation to temporary housing, no clear natural indirect effect estimate was observed. Meanwhile, the directionality was the same as that of the main result (Table 2). The RR for the natural direct effect estimate was 2.09 [95% CI: 1.15–3.33], which was also in the same direction as the main result. Likewise, for relocation to other types of housing, no clear associations were observed.

Similarly, the sensitivity analysis of the potential alternative mediator revealed a change in the frequency of group participation (Additional Table 5 [see Additional file 1]); the same directionalities were observed as the main analysis (Table 2), even though the natural indirect effect estimates for relocation to temporary housing were unclear. Likewise, the RR of the natural direct effect estimates of relocation to temporary housing was 3.41 [95% CI: 1.48–6.16] for MDE and 2.09 [95% CI: 1.39–3.45] for depression (GDS ≥ 5). The RR for PTSD symptoms and relocation to other housing types were unclear.

## Discussion

Renewal of group participation status, regardless of ceasing or starting after the 2016 Kumamoto earthquake, may have indirectly lowered the risk of MDE for those who relocated to temporary housing. Conversely, relocation to temporary housing itself may have directly enhanced the risk of MDE. No clear associations were observed for the outcome of PTSD symptoms or relocation to other housing types.

Contrary to our hypotheses, not all relocation type was directly associated with mental health issues, and changes in group participation were not stressors for older adults affected by disaster. Rather, for those who relocated to temporary housing, renewal of group participation status served as a stress reliever against MDE. We could not compare the results between relocation types, as no clear direct or indirect effects were observed for those who relocated to other housing types.

Moreover, renewal of group participation status may have been more beneficial to those who were severely depressed than those who were moderately depressed. We observed a clear indirect effect that relieved the risk of MDE (Table 2), but this association was not clear for depression measured as GDS ≥ 5 (Additional Table 4 [see Additional file 1]). Depression measured using the GDS included moderately depressed status with a score ≥ 5, regardless of symptom types. In contrast, the SQD rigorously measured MDE after the disaster; the scores were not only higher than the cutoff point but also included either depressed mood or diminished interest. Therefore, the MDE measured using SQD may have been limited to severe depression status, while GDS ≥ 5 may have included moderately depressed status. Therefore, it is reasonable that compared to those who were moderately depressed, those with a high risk of severe depression were more likely to be detected and protected by life supporters dispatched to temporary housing. Thus, those with a risk of MDE may have specifically benefited from optimizing group participation status with relief based on efforts by life supporters, such as promoting events at gathering places and linking them to mental health professionals.

Our results were consistent with previous studies reporting that relocation to temporary housing after earthquakes was associated with an increased risk of depression [10, 11]. However, in contrast to another study [20], our results showed that relocation to temporary housing (group relocation) had a clear positive association with ceased group participation. Moreover, utilizing causal mediation analysis and longitudinal data comparing relocated and non-relocated people, we reinforced the findings of a cross-sectional study of relocated people after the Great East Japan Earthquake of 2011. The findings showed that group participation was a more important factor against depression for those who relocated to temporary housing compared to those who relocated to rented housing [67]. Furthermore, this study elucidated that renewal of group participation status was a mediator which may relieve relocation stress against depression in temporary housing. At the temporary housing, group relocation policy, accessible public gathering places, and life supporters dispatched from the support center may have contributed to mitigating depression risk via renewal of group participation. Owing to the administrative efforts after the 2016 Kumamoto earthquake, the temporary housing might have offered opportunities for group participation in public gathering places and enabled people to strengthen their social ties with acquaintances [21] brought by group relocation or to develop social ties with new people [24]. Thus, temporary housing residents might have optimized their social ties or connections with others, as those who wished to belong to a group might have found a new one, while those who felt burdened with group membership or activities might have left them after the relocation [68, 69]. For the latter, the relocation might be an opportunity for optimizing social relationships, ending and/or reducing unwanted group participation. Even after quitting groups, they might have been protected by the temporary housing environment, where familiar residents lived nearby and life supporters visited, providing opportunities for social interactions. Therefore, some residents in temporary housing who felt uneasy with participating in social groups might have quit without the concern of losing social interactions.

Regarding relocation to other housing types, our results were consistent with those of previous studies where no clear associations with depression were observed [10, 11]. A potential explanation may be that some people who relocated to other types of housing, such as new private housing, might have been able to find accommodation more suited to their wishes. Thus, they might have experienced lower psychological burdens compared to those who relocated to temporary housing [70].

In contrast, for any type of housing, relocation was not associated with PTSD symptoms. This may be because we adjusted for property loss as a covariate because it was one of the traumatic events that reminded residents of the disaster, as reported in previous studies [18, 71]. Thus, effects of relocation on PTSD symptoms might be weakened after this adjustment.

This study leveraged a unique pre-post disaster dataset to overcome issues of using post-disaster data only [72] and clarified the mediator between relocation to temporary housing and MDE. To the best of our knowledge, this is the first longitudinal study to elucidate that renewal of group participation may mediate and alleviate the increased risks of MDE after relocation to temporary housing.

However, this study has several limitations. First, as mental health outcomes in this study were not based on diagnosis but measured by screening tools instead of interviews, the prevalence may be overestimated. Second, although we hypothesized that relocation, changes in group participation, and the onset of mental health issues occurred sequentially, the latter two were measured at the same wave owing to data restrictions. Thus, the existence of reverse causality cannot be ruled out. Third, we could not specify the types and exact state of group participation due to a lack of information. In our main analysis, we assumed that the change in status of ceasing or starting group participation (canceling or obtaining memberships to any group) occurred only after the earthquake. However, as the cutoff for the group participation mediator is a “few times a year,” the mediator may still include status changes that occurred within the pre-disaster period. To avoid possible temporal inconsistencies, we adopted a different cutoff criterion, “once a month,” for group participation in the sensitivity analysis (Additional Table 5 [see Additional file 1]). However, the mediating relationships remained unclear. Although status changes before the earthquake were rare, our main analysis may contain some temporal inconsistencies. Fourth, we could not distinguish between the effects of the physical and social environments of housing owing to data restrictions. Thus, the types of relocation (relocation to temporary housing vs. other types of housing) may reflect differences between both social environments (group vs. individual relocation) and physical environments (types of housing). The work of the support center and life supporters may also be reflected in the social environment of temporary housing. Fifth, the dataset may reflect selective attrition of people with depression (Additional Table 6 [see Additional file 1]), and the effects on mental health may be underestimated because of selection bias [73]. Nonetheless, our results showed a clear association between relocation to temporary housing and MDE. Sixth, although previous studies indicated gender differences in the effect of group participation on mental health [74, 75], in this study, mediation analysis stratified by gender did not converge, possibly because of the small sub-sample sizes. Even though we imputed the variables via MICE to utilize the entire available data of respondents, the overall sample size may have been small because the estimates still had large SEs, and the efficiencies of the estimates were not high. Seventh, in our results, some natural indirect effect estimates showed the opposite directionality to the total effect estimates. Thus, the calculation of the proportion mediated would not be appropriate in these cases. It should be noted that the use of the proportion mediated measures is desirable only when the directionality of the effect estimates is the same [56]. Eighth, against our assumption, there could be unmeasured confounding among the exposure, mediator, and outcomes or unmeasured exposure-induced mediator-outcome confounders. Ninth, the sample size may have been small to obtain robust estimates. However, a simulation study [76] that compared mediation analyses using five software showed that, compared with other methods, the inverse odds ratio-weighted method used in our study was relatively robust to bias based on the small sample size. Future studies are needed to investigate the long-term direct and indirect associations between relocation and the mental health of disaster-affected people using three waves of data.

## Conclusions

Disaster-related relocation to temporary housing may have a negative impact on MDE but may be mitigated via renewal of group participation. Older adults in temporary housing might have optimized their social ties, and that protected them against MDE in the short term. The process might have been supported by administrative efforts after the 2016 Kumamoto earthquake, such as a group relocation policy, accessible public gathering places, and dispatching life supporters from a support center. Older adults who relocated to temporary housing might have been able to choose a group participation status that they were comfortable with; even without any participation, they might be surrounded by familiar residents who lived nearby, thus maintaining their level of social interaction. Further studies are required to examine the potential long-term impacts of relocation to protect the mental health of people affected by disaster.

### Electronic supplementary material

Below is the link to the electronic supplementary material.

Supplementary Material 1

## Data Availability

All inquiries concerning the data are to be addressed to the data management committee via email: dataadmin.ml@jages.net. All Japan Gerontological Evaluation Study datasets have ethical or legal restrictions for public deposition owing to the inclusion of sensitive information from the human participants.

## Declarations

All participants were informed that participation in the study was voluntary and that completing and returning the questionnaire via mail indicated their consent to participate in the study. For any participants without written declaration of intention, written informed consent was assumed with the voluntary return of the questionnaire. The ethics committees approved the use of assumed consent upon return of the questionnaire. With this procedure, informed consent was obtained from all the participants involved in the study.

## Abbreviations

CI: confidence interval
GDS: Geriatric Depression Scale
GDS-15: 15-item Geriatric Depression Scale
JAGES: Japan Gerontological Evaluation Study
MDE: major depressive episodes
MICE: multiple imputation by chained equation
PTSD: posttraumatic stress disorder
RR: relative risk
SQD: Screening Questionnaire for Disaster Mental Health
SQD-D: Screening Questionnaire for Disaster Mental Health and six items on major depressive episodes
SQD-P: Screening Questionnaire for Disaster Mental Health and nine items on posttraumatic stress disorder
