# Supplementary material for: Does disaster-related relocation impact mental health via changes in group participation among older adults? Causal mediation analysis of a pre-post disaster study of the 2016 Kumamoto earthquake
Source: BMC Public Health. 2023 Oct 11;23:1982. doi: 10.1186/s12889-023-16877-0 (PMC10568925; doi:10.1186/s12889-023-16877-0)
Supplement: Supplementary file 1 — Supplementary Material 1 [file 12889_2023_16877_MOESM1_ESM.docx]

**Web Materials:**

**Does disaster-related relocation impact mental health via changes in group participation among older adults? Causal mediation analysis of a pre-post disaster study of the 2016 Kumamoto earthquake**

Additional Figure 1. Population density of Mifune Town, Kumamoto Prefecture in Japan (2013–2016). Point A is the epicenter of the Kumamoto earthquake on April 14, 2016. Point B is the epicenter of the Kumamoto earthquake on April 16, 2016 [1]. SD is the standard deviation.


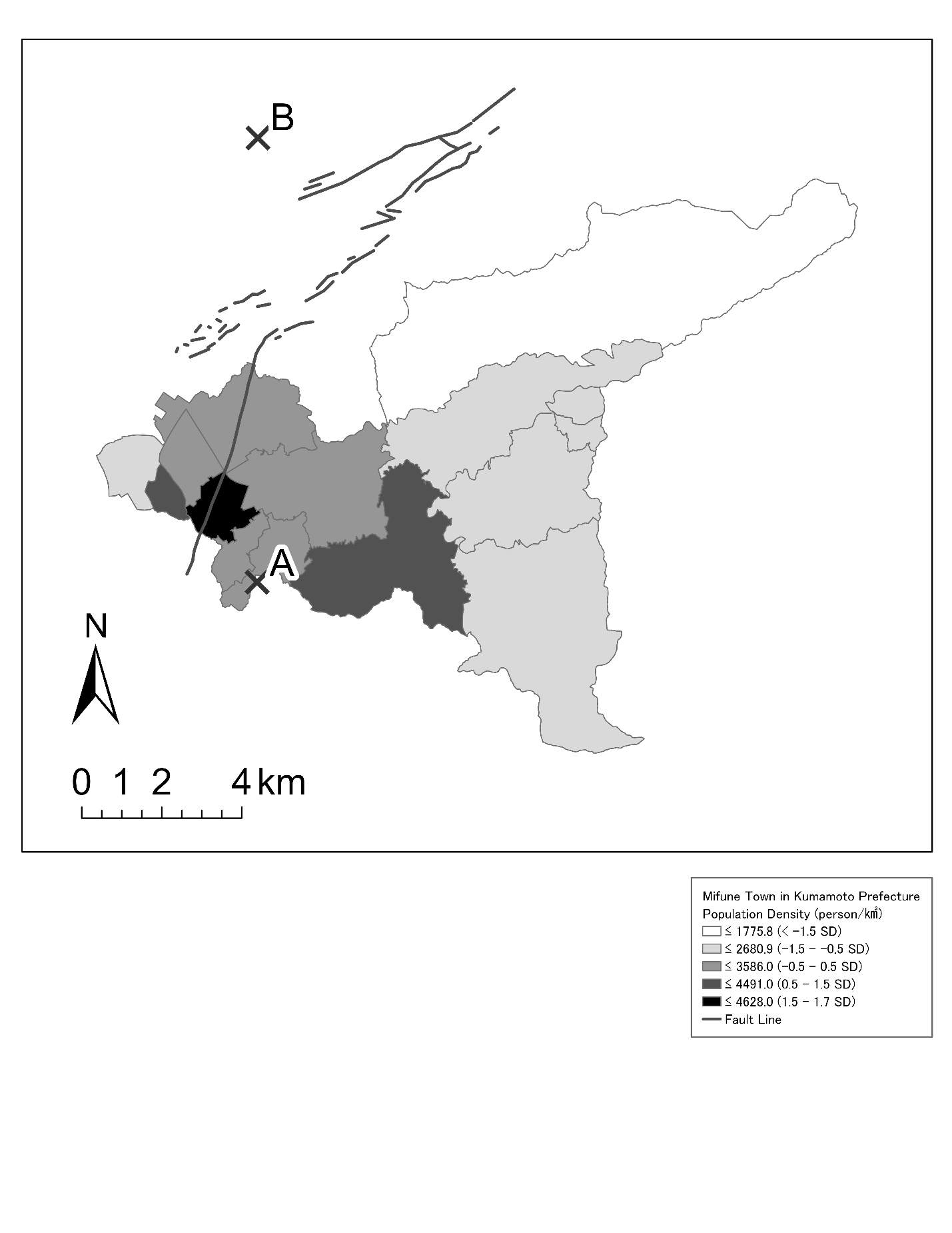


Additional Figure 2. Area slope of Mifune Town, Kumamoto Prefecture in Japan (2013–2016). Point A is the epicenter of the Kumamoto earthquake on April 14, 2016. Point B is the epicenter of the Kumamoto earthquake on April 16, 2016 [1]. SD is the standard deviation.


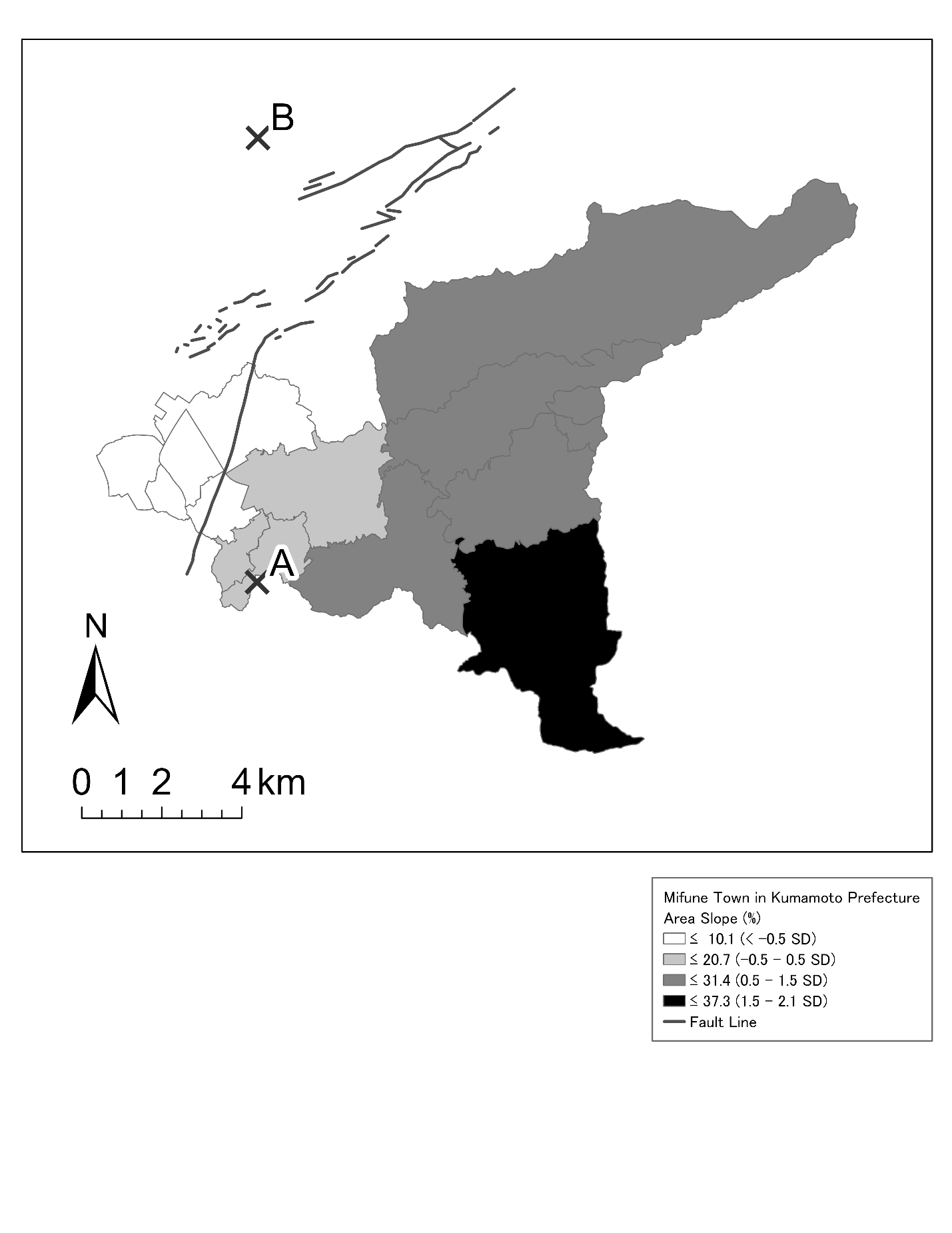


Additional Table 1. Relative risks with 95% confidence intervals: Results of first-leg analysis of multinomial logistic regression or Poisson regression with multiple imputation by chained equation for ceased, started, or renewed (ceased or started) group participation on types of relocation (n = 828), Mifune, Japan (2013–2016)

| Exposure (ref. no relocation) | Ceased  group participation | | | Started  group participation | | | Renewed  (ceased or started)  group participation | | |
| --- | --- | --- | --- | --- | --- | --- | --- | --- | --- |
|  | RR ^a^ | 95% CI ^c^ | | RR ^a^ | 95% CI ^c^ | | RR ^b^ | 95% CI ^c^ | |
| Relocation to temporary housing | 2.89 | 1.35 | 6.19 | 0.53 | 0.01 | 1.73 | 1.37 | 0.97 | 2.08 |
| Relocation to other types of housing | 10.32 | 3.21 | 32.22 | 0.12 | 0.00 | 0.73 | 2.14 | 1.43 | 3.85 |
| Abbreviations: RR, relative risk; CI, Confidence Interval; MICE, multiple imputation by chained equation | | | | | | | | | |
| ^a^ Result of multinomial regression: the outcome is ceased or started group participation (‘sustained’ as a reference) | | | | | | | | | |
| ^b^ Result of Poisson regression: the outcome is renewed (ceased or started) group participation (‘sustained’ as a reference) | | | | | | | | | |
| ^c^ Bootstrapped 1,000 times, 95% CI displays bias-corrected Confidence Interval | | | | | | | | | |
|  | | | | | | | | | |
| Dataset: Imputed dataset by MICE (m=20), including an exposure, a mediator, and covariates as imputed variables | | | | | | | | | |

Additional Table 2. Natural indirect effects, natural direct effects, and total effects estimates of the relocation on major depressive episodes via a single mediator (ceased or started group participation) by types of relocation: Mediation analysis by inverse odds weight from inverse odds ratio-weighted methods using Poisson regression with multiple imputation by chained equation (n = 828), Mifune, Japan (2013–2016)

|  | MDE | | | | | |
| --- | --- | --- | --- | --- | --- | --- |
|  | M1: ceased group participation ^a^ | | | M2: started group participation ^a^ | | |
|  | RR | 95% CI ^b^ |  | RR | 95% CI ^b^ |  |
| 1: Relocation to temporary housing |  |  |  |  |  |  |
| Natural Indirect Effect　(via ceased or started group participation) | 0.59 | 0.31 | 0.93 | 0.61 | 0.39 | 0.89 |
| Natural Direct Effect (of relocation) | 3.88 | 1.89 | 8.09 | 3.75 | 1.61 | 6.43 |
| Total Effect | 2.28 | 1.35 | 3.83 | 2.28 | 1.29 | 3.82 |
| 2: Relocation to other types of housing |  |  |  |  |  |  |
| Natural Indirect Effect　(via ceased or started group participation) | 0.98 | 0.62 | 1.89 | 1.16 | 0.73 | 2.36 |
| Natural Direct Effect (of relocation) | 1.60 | 0.61 | 3.04 | 1.36 | 0.45 | 2.64 |
| Total Effect | 1.58 | 0.90 | 2.68 | 1.58 | 0.94 | 2.74 |
| Abbreviations: MDE, major depressive episodes; RR, relative risk; CI, Confidence Interval; MICE, multiple imputation by chained equation | | | | | | |
| NOTE: Adjusted for the baseline covariates of gender, age, equivalent household income (< 2.0 million yen: low income), years of education (≤ 9 years of compulsory education indicated low education), lived alone or not, had an illness or not, had a job or not, with group participation or not, had depressive symptoms or not, standardized score of population density (persons/km^2^) and standardized score of area slope (%), and disaster damage in 2016 such as housing damage, and farmland damage | | | | | | |
| ^a^ Reference for M1 is ‘started or sustained,’ reference for M2 is ‘ceased or sustained.’ | | | | | | |
| ^b^ Bootstrapped 1,000 times, 95% CI displays bias-corrected Confidence Interval | | | | | | |
| Dataset: Imputed dataset by MICE (m=20), including an outcome (MDE), an exposure, a mediator, and covariates as imputed variables | | | | | | |

Additional Table 3. Natural indirect effects, natural direct effects, and total effects estimates of the relocation on post-traumatic stress disorder symptoms via a single mediator (ceased or started group participation) by types of relocation: Mediation analysis by inverse odds weight from inverse odds ratio-weighted methods using Poisson regression with multiple imputation by chained equation (n = 828), Mifune, Japan (2013–2016)

|  | PTSD symptoms | | | | | |
| --- | --- | --- | --- | --- | --- | --- |
|  | M1: ceased group participation ^a^ | | | M2: started group participation ^a^ | | |
|  | RR | 95% CI ^b^ |  | RR | 95% CI ^b^ |  |
| 1: Relocation to temporary housing |  |  |  |  |  |  |
| Natural Indirect Effect　(via ceased or started group participation) | 0.70 | 0.42 | 1.25 | 0.71 | 0.44 | 1.17 |
| Natural Direct Effect (of relocation) | 2.02 | 0.87 | 3.72 | 2.00 | 0.86 | 3.38 |
| Total Effect | 1.41 | 0.86 | 2.17 | 1.41 | 0.90 | 2.16 |
| 2: Relocation to other types of housing |  |  |  |  |  |  |
| Natural Indirect Effect　(via ceased or started group participation) | 0.96 | 0.58 | 1.32 | 1.25 | 0.93 | 2.13 |
| Natural Direct Effect (of relocation) | 1.56 | 0.87 | 2.76 | 1.21 | 0.51 | 1.93 |
| Total Effect | 1.50 | 1.00 | 2.22 | 1.50 | 0.97 | 2.17 |
| Abbreviations: PTSD, post-traumatic stress disorder; RR, relative risk; CI, Confidence Interval; MICE, multiple imputation by chained equation | | | | | | |
| NOTE: Adjusted for the baseline covariates of gender, age, equivalent household income (< 2.0 million yen: low income), years of education (≤ 9 years of compulsory education indicated low education), lived alone or not, had an illness or not, had a job or not, with group participation or not, had depressive symptoms or not, standardized score of population density (persons/km^2^) and standardized score of area slope (%), and disaster damage in 2016 such as housing damage, and farmland damage | | | | | | |
| ^a^ Reference for M1 is ‘started or sustained,’ reference for M2 is ‘ceased or sustained.’ | | | | | | |
| ^b^ Bootstrapped 1,000 times, 95% CI displays bias-corrected Confidence Interval | | | | | | |
| Dataset: Imputed dataset by MICE (m=20), including an outcome (PTSD symptoms), an exposure, a mediator, and covariates as imputed variables | | | | | | |

Additional Table 4. Natural indirect effects, natural direct effects, and total effects estimates of the relocation on depression measured by a 15-item Geriatric Depression Scale via a mediator of renewed (ceased or started) group participation by types of relocation: Mediation analysis by inverse odds weight from inverse odds ratio-weighted methods using Poisson regression with multiple imputation by chained equation (n = 828), Mifune, Japan (2013–2016)

|  | Depression (GDS ≥ 5) | | |  |
| --- | --- | --- | --- | --- |
|  | RR | 95% CI ^a^ |  |  |
| 1: Relocation to temporary housing |  |  |  |  |
| Natural Indirect Effect　(via renewed group participation) | 0.80 | 0.49 | 1.24 |  |
| Natural Direct Effect (of relocation) | 2.09 | 1.15 | 3.33 |  |
| Total Effect | 1.66 | 1.19 | 2.41 |  |
| 2: Relocation to other types of housing |  |  |  |  |
| Natural Indirect Effect　(via renewed group participation) | 0.94 | 0.69 | 1.33 |  |
| Natural Direct Effect (of relocation) | 1.80 | 0.97 | 3.28 |  |
| Total Effect | 1.69 | 1.10 | 2.80 |  |
| Abbreviations: GDS, 15-item Geriatric Depression Scale; RR, relative risk; CI, Confidence Interval; MICE, multiple imputation by chained equation | | | | |
| NOTE: Adjusted for the baseline covariates of gender, age, equivalent household income (< 2.0 million yen: low income), years of education (≤ 9 years of compulsory education indicated low education), lived alone or not, had an illness or not, had a job or not, with group participation or not, had depressive symptoms or not, standardized score of population density (persons/km^2^) and standardized score of area slope (%), and disaster damage in 2016 such as housing damage, and farmland damage | | | | |
| ^a^ Bootstrapped 1,000 times, 95% CI displays bias-corrected Confidence Interval | | | | |
| Dataset: Imputed dataset by MICE (m=20), including an outcome of depression (GDS ≥ 5), an exposure, a mediator, and covariates as imputed variables | | | | |

Additional Table 5. Natural indirect effect, natural direct effect, and total effect estimates of relocation on major depressive episodes, posttraumatic stress disorder symptoms, and depression, measured by the 15-item Geriatric Depression Scale, using change in frequency of group participation as a mediator (decreased or increased from once a month) based on the type of relocation: Mediation analysis by inverse odds weight from inverse odds ratio-weighted methods using Poisson regression with multiple imputation by chained equation (n = 828), Mifune, Japan (2013–16)

|  | MDE |  |  | PTSD symptoms | | | Depression (GDS ≥ 5) | | |
| --- | --- | --- | --- | --- | --- | --- | --- | --- | --- |
|  | RR | 95% CI ^a^ |  | RR | 95% CI ^a^ |  | RR | 95% CI ^a^ |  |
| 1: Relocation to temporary housing |  |  |  |  |  |  |  |  |  |
| Natural indirect effect (via group participation with a change in frequency) | 0.69 | 0.44 | 1.12 | 0.73 | 0.37 | 1.04 | 0.81 | 0.51 | 1.11 |
| Natural direct effect (of relocation) | 3.41 | 1.48 | 6.16 | 1.93 | 1.02 | 3.24 | 2.09 | 1.39 | 3.45 |
| Total effect | 2.35 | 1.29 | 3.82 | 1.40 | 0.91 | 2.20 | 1.69 | 1.21 | 2.52 |
| 2: Relocation to other types of housing |  |  |  |  |  |  |  |  |  |
| Natural indirect effect　(via group participation with a change in frequency) | 1.41 | 0.98 | 3.55 | 1.30 | 1.01 | 2.42 | 1.00 | 0.78 | 1.39 |
| Natural direct effect (of relocation) | 1.15 | 0.35 | 2.00 | 1.17 | 0.52 | 1.77 | 1.68 | 0.91 | 2.81 |
| Total effect | 1.62 | 0.88 | 2.70 | 1.52 | 1.00 | 2.30 | 1.68 | 1.08 | 2.68 |
| Abbreviations: MDE, major depressive episodes; PTSD, posttraumatic stress disorder; GDS, 15-item Geriatric Depression Scale; RR, relative risk; CI, confidence interval | | | | | | | | | |
| NOTE: Adjusted for the baseline covariates of gender, age, equivalent household income (< 2.0 million yen: low income), years of education (≤ 9 years of compulsory education indicated low education), lived alone or not, had an illness or not, had a job or not, with group participation or not, had depressive symptoms or not, standardized score of population density (persons/km^2^) and standardized score of area slope (%), and disaster damage in 2016 such as housing and farmland damage | | | | | | | | | |
| ^a^ Bootstrapped 1,000 times, 95% CI displays bias-corrected confidence interval | | | | | | | | | |
| Dataset: Imputed dataset by multiple imputation by chained equation (m = 20), including an outcome (MDE or PTSD symptoms or depression (GDS ≥ 5)), an exposure, a mediator, and covariates as imputed variables | | | | | | | | | |

Additional Table 6. Summary statistics of comparison between the participants included in the analyses (n = 828) and non-participants (n = 476) (before imputation), Mifune, Japan (2013–2016)

|  | Participants ^a^ (n = 828) | | | | Non-Participants ^b^ (n = 476) | | | |
| --- | --- | --- | --- | --- | --- | --- | --- | --- |
|  | n | % | Mean | SD | n | % | Mean | SD |
|  |  |  |  |  |  |  |  |  |
| COVARIATES |  |  |  |  |  |  |  |  |
| *Baseline personal characteristics*  *(before earthquake)* |  |  |  |  |  |  |  |  |
| Gender: |  |  |  |  |  |  |  |  |
| Female | 467 | 56.4 |  |  | 234 | 49.2 |  |  |
| Male | 361 | 43.6 |  |  | 242 | 50.8 |  |  |
| Age: |  |  |  |  |  |  |  |  |
| 65–69 | 230 | 27.8 |  |  | 77 | 16.2 |  |  |
| 70–74 | 259 | 31.3 |  |  | 116 | 24.4 |  |  |
| 75–79 | 173 | 20.9 |  |  | 113 | 23.7 |  |  |
| 80–84 | 112 | 13.5 |  |  | 93 | 19.5 |  |  |
| 85+ | 54 | 6.5 |  |  | 77 | 16.2 |  |  |
| Low income | 422 | 51.0 |  |  | 235 | 49.4 |  |  |
| Low education | 338 | 40.8 |  |  | 235 | 49.4 |  |  |
| Living alone | 88 | 10.6 |  |  | 69 | 14.5 |  |  |
| No illness | 108 | 13.0 |  |  | 47 | 9.9 |  |  |
| No job | 557 | 67.3 |  |  | 320 | 67.2 |  |  |
| No  Group participation  at baseline | 152 | 18.4 |  |  | 114 | 23.9 |  |  |
| Depressive symptoms at baseline: |  |  |  |  |  |  |  |  |
| Not depressed | 568 | 68.6 |  |  | 261 | 54.8 |  |  |
| Moderately depressed | 96 | 11.6 |  |  | 80 | 16.8 |  |  |
| Depressed | 25 | 3.0 |  |  | 29 | 6.1 |  |  |
| *Baseline regional characteristics*  *(before earthquake)* |  |  |  |  |  |  |  |  |
| Population density (person/km^2^) ^c^ | 828 |  | 3289.0 | 847.5 | 472 |  | 3240.6 | 881.1 |
| Area slope (%) ^c^ | 828 |  | 14.3 | 9.1 | 472 |  | 15.3 | 8.7 |
| Abbreviations: SD, standard deviation | | | | | | | | |
| ^a^ Participants: those who participated both the baseline survey in 2013 and the follow-up survey in 2016 | | | | | | | | |
| ^b^ Non-participants: those who participated only in the baseline survey in 2013 and did not participate in the follow-up survey in 2016 | | | | | | | | |
| ^c^ Variables are standardized after multiple imputations to avoid multicollinearity and for ease of interpretation | | | | | | | | |

Web Appendix 1. Outline of the inverse odds ratio-weighted (IORW) Method

The inverse odds ratio-weighted (IORW) method by EJ Tchetgen Tchetgen [2] has several strengths in mediation analysis. First, it allows for a wider flexibility for selection of regression models, and multiple mediators or any types of variables can be included in the models [3-5]. Second, interactions between an exposure and mediators were allowed because they were condensed into weights after controlling for covariates [3, 6]. Third, the model specification was practically more feasible because we only needed an assumption of exposure variable distribution [5].

Web Appendix 2. Inverse odds weighted mediation analysis for 3-level exposures

We referred to the practical guidance of QC Nguyen, TL Osypuk, NM Schmidt, MM Glymour, and EJ Tchetgen Tchetgen [3] and adopted inverse odds weight (IOW) for the analysis. Additionally, we augmented weight calculation to a multinomial logistic regression [7] and derived weights for 3-level categorical exposure variable. Calculating the total effect, the natural direct effect, and the natural indirect effect estimates was based on the causal mediation formula by J Pearl [8] and J Pearl [9]. Based on the assumption of missing at random (MAR), we used 20 imputed data sets produced by multiple imputation by chained equation (MICE).

Based on the practical guidance [3] (from step 1 to 5, with some modifications), we estimated the total effect (TE), the natural direct effect (NDE), and the natural indirect effect (NIE) using the following steps:

1) We calculated inverse odds weight (IOW) by multinomial logistic regressions of an exposure (X) on a mediator (M) and covariates (C).

2) We estimated the total effect from unweighted Poisson regressions of an outcome (Y) on an exposure (X) and covariates (C).

3) Additionally, we used IOW and estimated the natural direct effect from weighted Poisson regressions of an outcome (Y) on an exposure (X) and covariates (C).

4) Next, for the effect decomposition by log-scale calculation, we subtracted the coefficient of the natural direct effect estimate from the coefficient of the total effect estimate and derived the coefficient of the natural indirect effect estimate.

(i.e., $\log NIE= \log TE- \log NDE$)

5) Thereafter, we bootstrapped the effect estimates for 1,000 times and derived a bias-corrected 95% confidence interval for the estimates of the natural indirect effect, the natural direct effect, and the total effect.

Web Appendix 3. STATA code for inverse odds weighted mediation analysis for 3-level exposures

STATA code example based on;

QC Nguyen, TL Osypuk, NM Schmidt, MM Glymour and EJ Tchetgen Tchetgen [3] WEB APPENDIX 3

NOTE: **bold** sections are revised for treating 3-level exposures

------------------------------------------------------------------------------------------------------

*define a user-written program;

capture program drop IOWMICE

program IOWMICE, rclass

*tell Stata to preserve the data. Data are restored after program termination ;

preserve

*Insert equation to impute missing data. We request 20 imputed datasets ;

ice outcome mediator covariate1 covariate2 , saving(“filelocation\nameofdataset.dta” , replace) m(20)

use “filelocation\nameofdataset.dta” , clear

*Retain estimates of predicted probability, inverse odds, and inverse odds weights for later use ;

capture drop logodds**1 logodds2** predprob**1 predprob2** inverseodds**1 inverseodds2** wt_iow

*Insert regression of treatment on mediator and covariates. Mim command analyzes multiply imputed data. Storebv command stores regression results for later use;

mim, storebv: **m**logit treatment mediator covariate1 covariate2**, base(0)**

*Calculate predicted log odds and use that to calculate predicted probabilities and inverse odds;

predict logodds**1**, xb **outcome(1)**

**predict logodds2, xb outcome(2)**

gen predprob**1** = exp(logodds**1**)/(1+exp(logodds**1**)**+ exp(**logodds**2))**

gen inverseodds**1** = ((1-predprob**1**)/predprob**1**)

**gen predprob2 = exp(logodds2)/(1+exp(logodds1)+exp(logodds2))**

**gen inverseodds2 = ((1-predprob2)/predprob2)**

*Calculate inverse odds weights;

gen wt_iow = 1 if treatment==0

replace wt_iow = inverseodds**1** if **treatment==1**

**replace wt_iow = inverseodds2 if treatment==2**

* Insert the total effect regression here and retain estimate of total effects for later use ;

mim, storebv: glm outcome **i.**treatment covariate1 covariate2, fam(poisson) link(log) vce(robust)

**matrix bb_total_1= e(b)**

**scalar b_total_1=bb_total_1[1,2]**

**return scalar b_total_1=bb_total_1[1,2]**

**matrix bb_total_2= e(b)**

**scalar b_total_2=bb_total_2[1,3]**

**return scalar b_total_2=bb_total_2[1,3]**

* Insert the direct effect regression here. Retain estimate of direct effects. Calculate indirect effects as the difference between total effects and direct effects;

mim, storebv: glm outcome **i.**treatment covariate1 covariate2 [pweight=wt_iow], fam(poisson) link(log) vce(robust)

**matrix bb_direct_1 = e(b)**

**scalar b_direct_1=bb_direct_1[1,2]**

**return scalar b_direct_1=bb_direct_1[1,2]**

**return scalar b_indirect_1 = b_total_1-b_direct_1**

**matrix bb_direct_2 = e(b)**

**scalar b_direct_2=bb_direct_2[1,3]**

**return scalar b_direct_2=bb_direct_2[1,3]**

**return scalar b_indirect_2 = b_total_2-b_direct_2**

end

*Request bootstrapped estimates of indirect, direct and total effects. Provide initial value of the random-number seed so estimates can be replicated at a later time. Request 1000 bootstrap replications ;

bootstrap **r(b_indirect_1) r(b_direct_1) r(b_total_1) r(b_indirect_2) r(b_direct_2) r(b_total_2)**, seed(32222) reps(1000) : IOWMICE

estat bootstrap, all

estat bootstrap, eform all

**References**

1. GSI Maps. n.d. <https://www.gsi.go.jp/ENGLISH/index.html>. Accessed 16 Feb 2023
2. Tchetgen Tchetgen EJ. Inverse odds ratio-weighted estimation for causal mediation analysis. Stat Med*.* 2013;32(26):4567-80.
3. Nguyen QC, Osypuk TL, Schmidt NM, Glymour MM, Tchetgen Tchetgen EJ. Practical guidance for conducting mediation analysis with multiple mediators using inverse odds ratio weighting. Am J Epidemiol. 2015;181(5):349-56.
4. Schmidt NM, Nguyen QC, Kehm R, Osypuk TL. Do changes in neighborhood social context mediate the effects of the moving to opportunity experiment on adolescent mental health? Health Place. 2020;63:102331.
5. Starkopf L, Andersen MP, Gerds T, Torp-Pedersen C, Lange T: Comparison of five software solutions to mediation analysis. University of Copenhagen; 2017.
6. Schmidt NM, Glymour MM, Osypuk TL. Housing mobility and adolescent mental health: the role of substance use, social networks, and family mental health in the moving to opportunity study. SSM - Population Health. 2017;3:318-25.
7. Kleinbaum DG, Klein M: Polytomous Logistic Regression. In: Logistic Regression: A Self-Learning Text. edn. New York, NY: Springer New York; 2010:429-62.
8. Pearl J. Direct and indirect effects. In: Proceedings of the Seventeenth conference on Uncertainty in artificial intelligence*.* Seattle, Washington: Morgan Kaufmann Publishers Inc.; 2001: 411-20.
9. Pearl J. The causal mediation formula--a guide to the assessment of pathways and mechanisms. Prev Sci. 2012;13(4):426-36.
